# Experimental Study of Surface Reinforcement of Soil Slopes Using Carbon Fiber-Reinforced Stabilized Soil

**DOI:** 10.3390/ma19204375

**Published:** 2026-10-18

**Authors:** Sewook Oh, Sunghwan Yang, Donggeun Kim, Yoseok Jeong

**Affiliations:** 1 Department of Construction and Disaster Prevention Engineering, Kyungpook National University, Gyeongbuk 37224, Republic of Korea; osw@knu.ac.kr (S.O.); yang@syeway.co.kr (S.Y.); 2 Department of Forest Ecology and Protection, Kyungpook National University, Gyeongbuk 37224, Republic of Korea; dgkim96@knu.ac.kr

**Keywords:** carbon fiber reinforcement, soil stabilization, slope protection, artificial rainfall, erosion resistance, rainfall-induced deformation

## Abstract

Rainfall-induced erosion and deformation of soil slopes remain important concerns for long-term slope stability, highlighting the need for effective surface reinforcement methods. This study experimentally investigated the mechanical and rainfall-resistant performance of carbon fiber-reinforced stabilized soil for slope surface protection. Four soil types were initially evaluated using unconfined compression tests with different Eco Soil Binder (E.S.B.) contents, and well-graded sand (SW) with 20% E.S.B. was selected for further testing. Carbon fiber contents ranging from 0 to 1.2% were considered; however, stable specimens could not be formed at 1.2%, while 0.6% provided the highest compressive strength among the valid test conditions. The selected mixture of SW, 20% E.S.B., and 0.6% carbon fiber was further characterized through triaxial compression tests, yielding an apparent cohesion of 0.152 MPa and a friction angle of 37.23°. Artificial rainfall tests were then conducted using 1:50 geometrically scaled slope models with berm lengths of 0.05 and 0.15 m under a rainfall intensity of 20 mm/h for 48 h. Under the tested small-scale conditions, the stabilized slope models exhibited approximately 47–60% lower settlement than the unreinforced models, together with lower final moisture contents and less visible surface deterioration. Because the two model configurations differed in both material composition and initial unit weight, these results should be interpreted as descriptive comparative observations rather than as isolated effects of E.S.B. and carbon fiber.

## 1. Introduction

Recent climate change and the increasing frequency of extreme rainfall events have raised serious concerns regarding the stability of soil slopes. Intense or prolonged rainfall can infiltrate into near-surface soil layers, increase moisture content and pore-water pressure while reducing matric suction and shear strength. At the same time, surface runoff can detach and transport soil particles, resulting in erosion, scour, settlement, and progressive deformation of the slope surface. These processes are particularly critical in shallow soil slopes, where the surface zone is exposed to direct rainfall impact and generally experiences relatively low confining stress. Once localized erosion or deformation begins, progressive soil loss may further weaken the slope and eventually lead to larger-scale failure. Such rainfall-induced damage is a major concern for slopes associated with roads, embankments, and other geotechnical infrastructure [1,2,3]. Therefore, effective surface protection and reinforcement methods are required to improve the resistance of soil slopes against rainfall-induced erosion and deformation.

Soil stabilization has been widely used to improve the engineering performance of weak or problematic soils. Conventional chemical stabilization typically involves mixing in situ soil with cementitious or pozzolanic additives such as Portland cement, lime, and fly ash. These stabilizers can enhance unconfined compressive strength, shear strength, stiffness, and durability, while reducing plasticity, swelling potential, deformation, and permeability [4,5,6,7,8,9]. The improvement in stabilized soil is mainly attributed to hydration and subsequent pozzolanic reactions between the stabilizer, water, and soil constituents. In cement-based stabilization, hydration produces cementitious compounds such as calcium silicate hydrate (C–S–H) and calcium aluminate hydrate (C–A–H), which bind soil particles and form a denser and stronger matrix [10]. Secondary pozzolanic reactions generate additional cementitious products, further strengthening interparticle bonding and reducing pore spaces [8]. Consequently, stabilized soils generally exhibit improved compressive and shear resistance as well as greater resistance to moisture-induced deterioration, suggesting their potential use as materials for slope surface reinforcement and protection.

Despite the substantial improvements in strength and stiffness achieved through cement stabilization, cement-treated soils often exhibit relatively low tensile and flexural resistance and brittle failure characteristics, which can lead to rapid crack propagation after peak loading [9,11,12]. To overcome these limitations, various types of fibers have been incorporated into stabilized soils to enhance tensile and flexural strength, crack resistance, toughness, and ductility [13,14,15]. Previous studies have shown that both natural and synthetic fibers can effectively improve the mechanical behavior of soils and stabilized soils. Natural fibers, including palm and sisal fibers, have been reported to increase shear strength and ductility [16,17,18], whereas synthetic fibers have been shown to improve unconfined compressive strength, shear resistance, and post-peak behavior [19,20,21]. These improvements are generally attributed to the crack-bridging and fiber–soil interaction mechanisms, which restrain crack propagation, improve residual strength, and transform brittle failure into a more ductile response [22,23].

Previous studies have demonstrated that carbon fiber reinforcement can improve the compressive, shear and deformation characteristics of soils through fiber–soil interfacial bonding and friction [24,25,26]. More recently, chopped carbon fibers have been shown to enhance the flexural strength of soil–cement and transform the brittle failure into a more ductile post-peak behavior [27]. However, most previous studies have focused primarily on material-scale mechanical performance, using laboratory tests such as unconfined compression, tensile, flexural, direct shear, and triaxial tests. Consequently, although the mechanical benefits of carbon fiber reinforcement have been reasonably established, its effectiveness at the slope scale under rainfall conditions have received comparatively limited attention. In particular, limited experimental evidence is available regarding how carbon fiber-reinforced stabilized soil affects rainfall-induced settlement, moisture accumulation, surface erosion, and progressive failure. Therefore, an important research gap remains in linking the improved mechanical properties observed at the material scale with the actual reinforcement performance of soil slopes subjected to prolonged rainfall. In order to address this gap, this study evaluates carbon fiber-reinforced stabilized soil through mechanical tests and artificial rainfall model tests, comparing reinforced and unreinforced slopes in terms of settlement, moisture content, surface erosion, and failure behavior. By integrating material-scale mechanical characterization with slope-scale rainfall testing, this study provides an experimental basis for evaluating the applicability of carbon fiber-reinforced stabilized soil to rainfall-resistant slope surface protection.

## 2. Experimental Program

### 2.1. Materials

Four soil types with different engineering characteristics were investigated in this study and classified according to the Unified Soil Classification System (USCS) as well—graded sand (SW), poorly graded sand (SP), clayey sand (SC), and low-plasticity clay (CL), as summarized in Table 1. The maximum dry unit weight decreased from 21.0 kN/m^3^ for SW to 15.5 kN/m^3^ for CL, whereas the optimum moisture content generally increased with increasing fine-grained characteristics, ranging from 12 to 14% for the sandy soils to 40% for CL. SW and SP exhibited relatively low plasticity, while SC and CL showed higher plasticity indices of 11.3 and 16.0, respectively. The differences in gradation, compaction characteristics, and plasticity provided a basis for evaluating the applicability of the E.S.B. (Eco Soil Binder) stabilizer to soils with different engineering properties.

**Figure 1:**
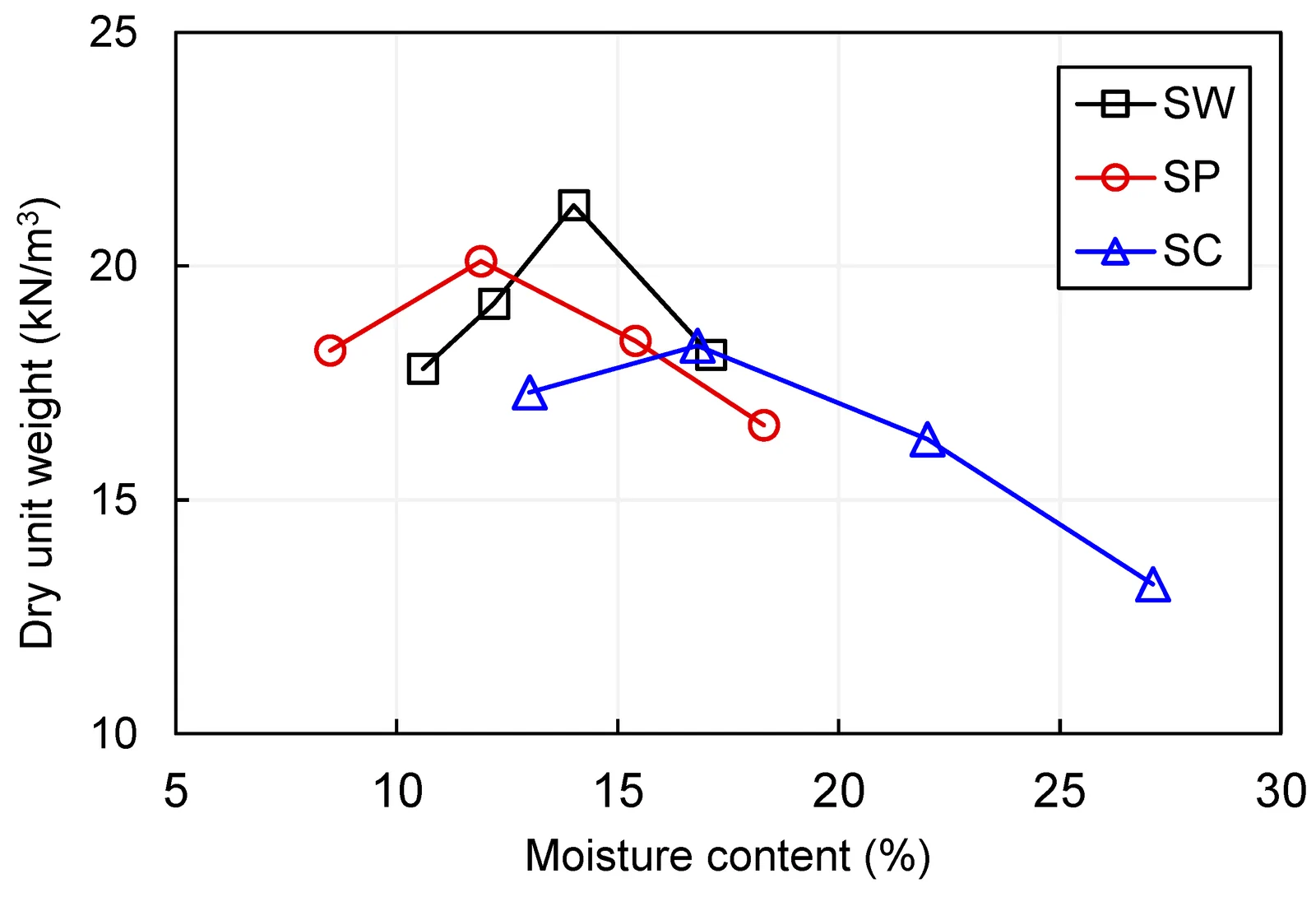
Compaction characteristics of the SW, SP, and SC soils.

**Figure 2:**
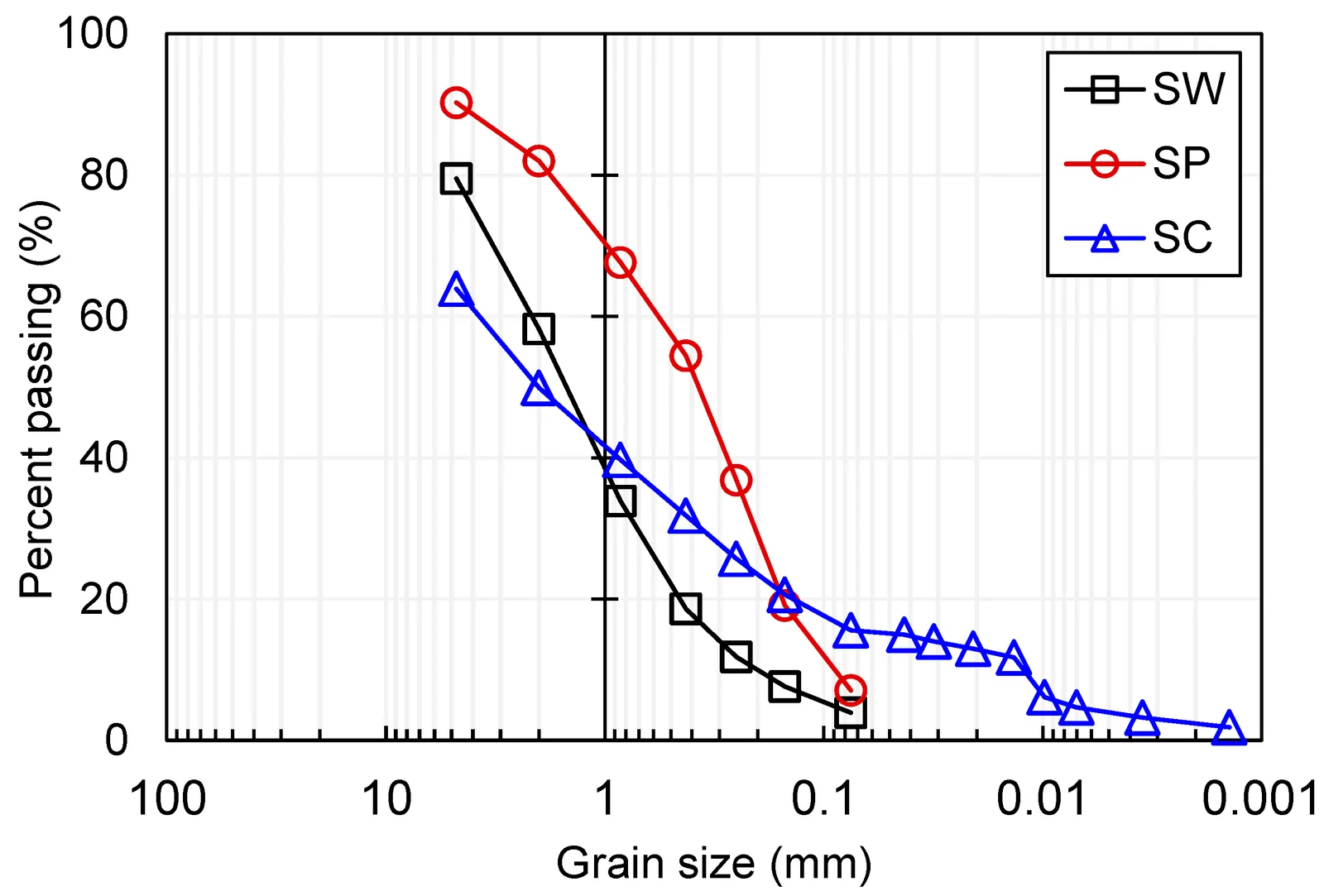
Grain-size distributions of SW, SP, and SC soils.

Figure 1 presents the compaction characteristics of the SW, SP, and SC soils. The maximum dry unit weights of SW, SP, and SC were 21.0, 19.2, and 18.0 kN/m^3^, respectively, with corresponding optimum moisture contents of 14%, 12%, and 18%. SW exhibited the highest maximum dry unit weight, whereas SC showed a lower maximum dry unit weight and a higher optimum moisture content. These differences reflect variations in soil gradation and fines content and were considered in determining the moisture and compaction conditions for specimen preparation. The compaction parameters of CL, which exhibited a substantially higher optimum moisture content of 40%, are summarized in Table 1. Figure 2 presents the grain-size distributions of the SW, SP, and SC soils, showing clear differences in their gradation characteristics. The consistency properties of CL, including a liquid limit of 42% and a plasticity index of 16, are also provided in Table 1.

Figure 2 presents the grain-size distributions of the SW, SP, and SC soils. SW exhibited a relatively broad particle-size distribution, consistent with its classification as well-graded sand, whereas SP showed a comparatively narrower gradation, corresponding to poorly graded sand. SC contained a larger proportion of fine particles than SW and SP, reflecting its clayey sand classification. These differences in particle-size distribution are consistent with the coefficients of uniformity and curvature summarized in Table 1 and indicate distinct gradation characteristics among the soils. The variation in gradation was considered an important factor affecting compaction behavior and the subsequent mechanical response of the stabilized soils.

Table 2 summarizes the chemical and physical properties of the Eco Soil Binder (E.S.B.) used in this study and compares them with those of conventional cement. E.S.B. contained a higher CaO content (63.08%) than cement (40.6%), while its SiO_2_ and Al_2_O_3_ contents were 20.07% and 5.21%, respectively. The specific surface area of E.S.B. was 3770 cm^2^/g, which was higher than that of cement (2800 cm^2^/g), indicating a relatively finer material. The E.S.B. also exhibited substantially shorter initial and final setting times of 50 and 75 min, respectively, compared with 260 and 465 min for cement. In contrast, the heat of hydration of E.S.B. was 12.59 cal/g, considerably lower than the 39 cal/g measured for cement. These characteristics indicate that E.S.B. provides a distinct chemical composition and setting behavior compared with conventional cement.

The carbon fiber used in this study was a 6K-grade product manufactured by HYOSUNG Advanced Materials [28]. The manufacturer-reported mechanical properties are summarized in Table 3. The carbon fiber had a tensile strength of 2900 MPa, a tensile modulus of 140 GPa, and a tensile strain of 1.8%. Its flexural modulus and interlaminar shear strength were reported as 125 GPa and 87 MPa, respectively. These properties indicate the high tensile stiffness and strength of the carbon fiber, which are advantageous for improving load transfer and restraining crack development when incorporated into stabilized soil. The fibers were cut to the prescribed length before mixing with the soil–binder matrix.

### 2.2. Specimen Preparation and Mechanical Testing

**Figure 3:**
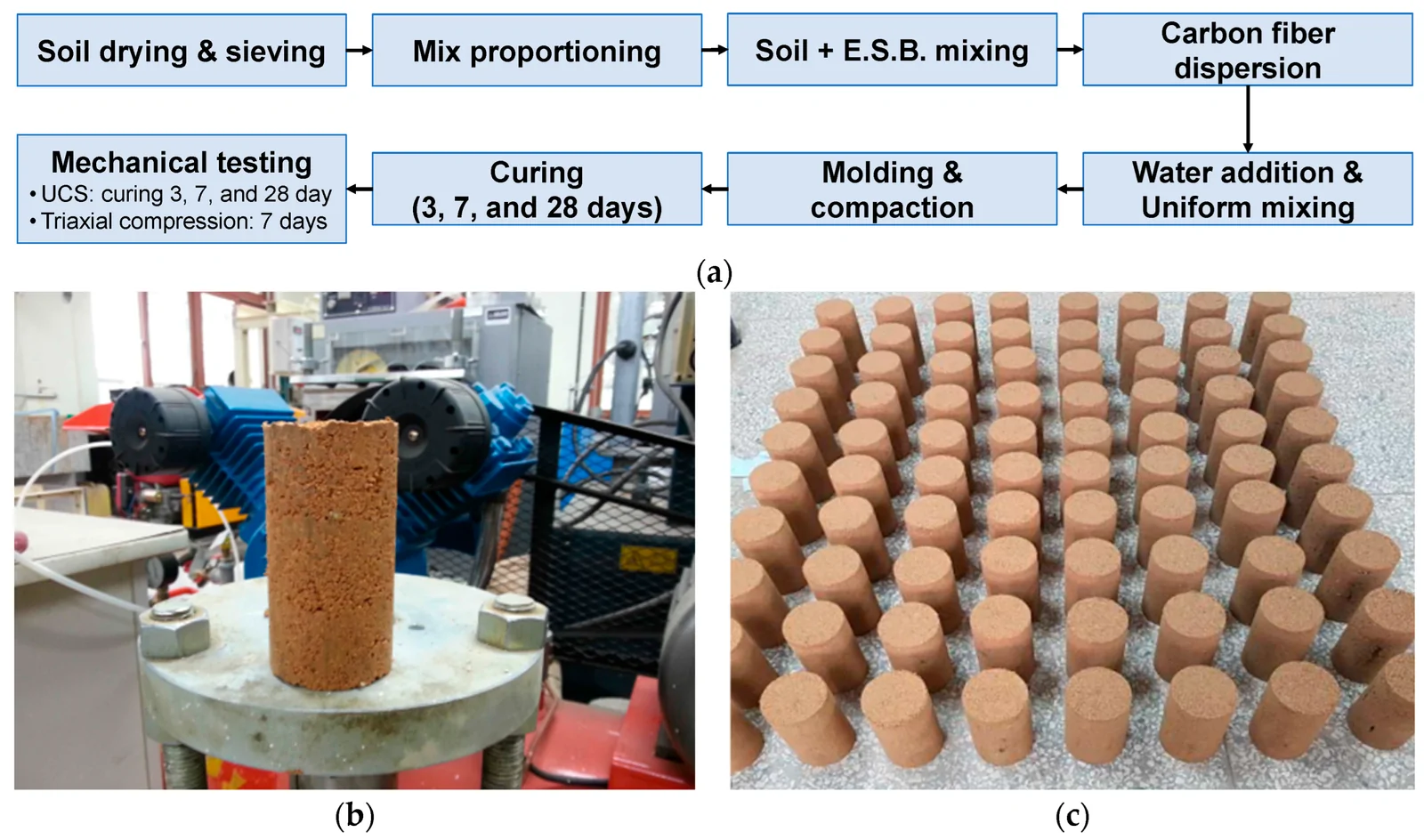
Specimen preparation: (**a**) flow chart of preparing specimens, (**b**) preparation process, and (**c**) specimens for unconfined compression tests.

Mechanical tests were conducted to evaluate the strength and deformation characteristics of the stabilized soils and to determine the mixture suitable for subsequent slope model tests. The experimental program consisted of unconfined compression, direct shear, and triaxial compression tests. Specimens for the unconfined compression and triaxial tests were prepared by thoroughly mixing the soil, Eco Soil Binder (E.S.B.), carbon fiber, and water at the prescribed proportions, followed by compaction into cylindrical molds. The prepared specimens were cured under laboratory conditions for the specified curing periods prior to testing. Figure 3 shows the specimen preparation process and the cylindrical specimens prepared for the unconfined compression tests.

#### 2.2.1. Unconfined Compression Tests

Unconfined compression tests were conducted in accordance with KS F 2314, which is comparable to ASTM D2166/D2166M, to evaluate the effects of E.S.B. and carbon fiber contents on the mechanical performance of the stabilized soils [29,30]. Cylindrical specimens with a diameter of 50 mm and a height of 100 mm were prepared at a relative compaction of 95% of the maximum dry unit weight. The soil, E.S.B., carbon fiber, and water corresponding to the optimum moisture content were thoroughly mixed, and the mixture was compacted into the mold in three layers to obtain uniform specimens. After curing under laboratory conditions for the specified periods, the specimens were tested using a universal testing machine under strain-controlled loading at a rate of 1%/min. For each test condition, three replicate specimens were prepared and tested, and the mean value of the three UCS measurements was used for subsequent analysis.

The UCS test program was evaluated in two steps, as summarized in Table 4. First, the effects of soil type and E.S.B. content were examined for SW, SP, SC, and CL at E.S.B. contents of 10%, 20%, and 30% to identify a suitable soil–binder combination. Based on this screening, SW stabilized with 20% E.S.B. was selected for evaluating the effect of carbon fiber reinforcement. Carbon fiber contents of 0, 0.3%, 0.6%, 0.9%, and 1.2% were considered using 6 mm long chopped fibers. Curing periods of 3, 7, and 28 days were used to evaluate strength development with time. This evaluation procedure was used to determine the mixture adopted for the subsequent triaxial compression and artificial rainfall model tests.

#### 2.2.2. Direct Shear Tests

Direct shear tests were conducted in accordance with KS F 2343, which is comparable to ASTM D3080/D3080M, to determine the shear strength parameters of the field soil used for the slope model tests [31,32]. The field soil was classified as well-graded sand (SW), and specimens were prepared with a diameter of 60 mm and a height of 30 mm. The specimens were compacted in three layers, with 15 compaction blows applied to each layer. To investigate the influence of moisture condition on shear resistance, three soil conditions were considered: dry, wet, and saturated. The corresponding unit weights were 18.4, 20.5, and 21.5 kN/m^3^, respectively, while moisture contents of 9.0% and 16.6% were applied to the wet and saturated specimens, respectively.

Normal stresses of 0.2, 0.4, and 0.6 MPa were applied to each specimen, as summarized in Table 5. Prior to shearing, each normal stress was maintained for approximately 10 min to allow the applied stress to be uniformly transferred to the specimen. The specimens were subsequently sheared at a constant displacement rate of 0.1 mm/min to a horizontal displacement corresponding to approximately 15% of the specimen diameter. The peak shear stresses obtained under the three normal-stress levels were used to establish the Mohr–Coulomb failure envelope and determine the cohesion, *c*, and friction angle, *ϕ*, of the SW soil under each moisture condition.

#### 2.2.3. Triaxial Compression Tests

Triaxial compression tests were conducted to determine the shear-strength parameters of the carbon fiber-reinforced stabilized soil. Based on the unconfined compression test results, SW stabilized with 20% E.S.B. and 0.6% carbon fiber was selected as the representative mixture for the triaxial tests. Cylindrical specimens with a diameter of 50 mm and a height of 100 mm were prepared using the same procedure as that adopted for the unconfined compression tests and were cured for 7 days before testing. Since the stabilized specimens exhibited a relatively rigid and cemented behavior, the triaxial testing procedure was conducted with reference to ASTM D2664-04 for confined compression testing of rock-like materials [33].

As summarized in Table 6, confining pressures (σ_3_) of 0.2, 0.4, and 0.6 MPa were applied, and axial loading was imposed under strain control at a rate of 1%/min. The axial load was continuously recorded until failure, and the post-peak response was monitored up to an axial strain of approximately 5%. The principal stresses obtained at failure under the three confining pressures were used to construct Mohr stress circles and the corresponding Mohr–Coulomb failure envelope. The apparent cohesion, c, and friction angle, ϕ, were determined from the Mohr–Coulomb failure envelope obtained under the present triaxial test conditions.

### 2.3. Artificial Rainfall Model Test

**Figure 4:**
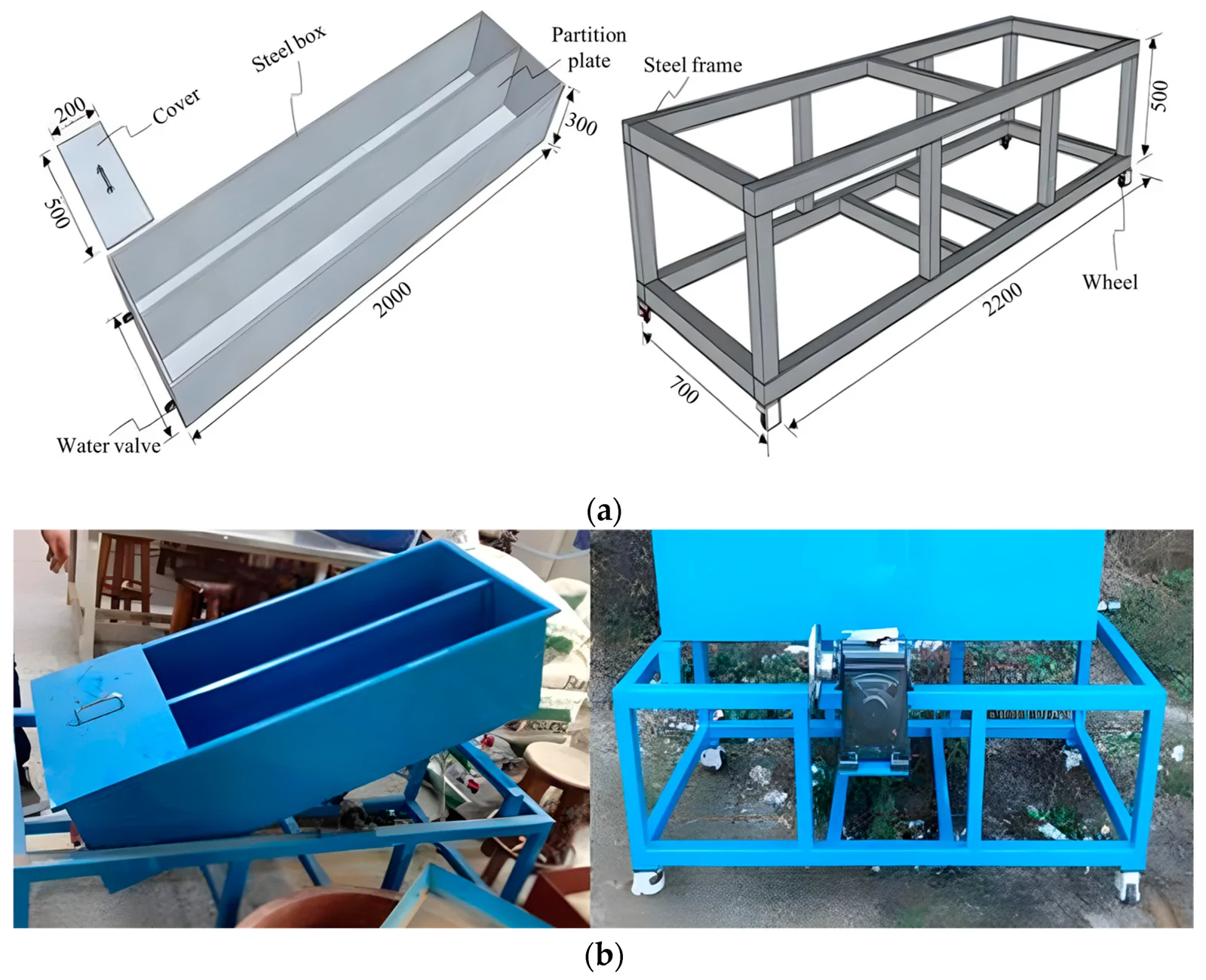
(**a**) Schematic and (**b**) overall view of the indoor artificial rainfall model test apparatus.

**Figure 5:**
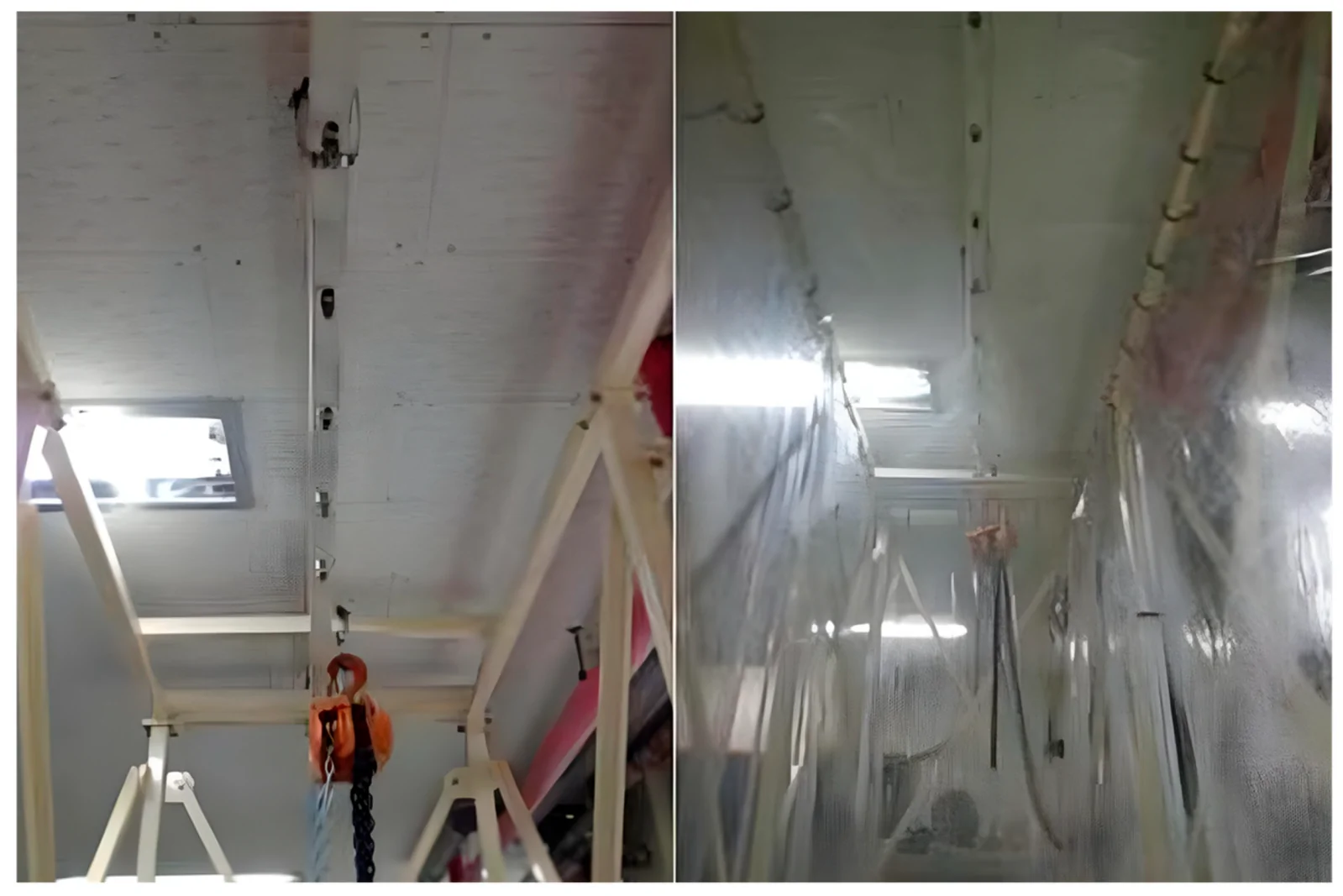
Rainfall control and distribution apparatus used in this study.

Indoor artificial rainfall model tests were conducted to compare the rainfall-induced responses of the unreinforced and locally stabilized slope models. A geometrically scaled model corresponding to 1:50 of the prototype slope was constructed using the apparatus shown in Figure 4. The 6 mm carbon fibers used in the stabilized soil were not geometrically scaled with the slope model; therefore, complete similitude of the fiber–soil reinforcement mechanism was not achieved. The test system consisted of a steel soil box mounted on a supporting steel frame and an overhead rainfall control and distribution system. The rainfall system was designed to provide a continuous and approximately uniform water supply over the model slope during testing. Figure 4a,b presents the schematic configuration and overall view of the test apparatus. Figure 5 shows the rainfall control and distribution system.

**Figure 6:**
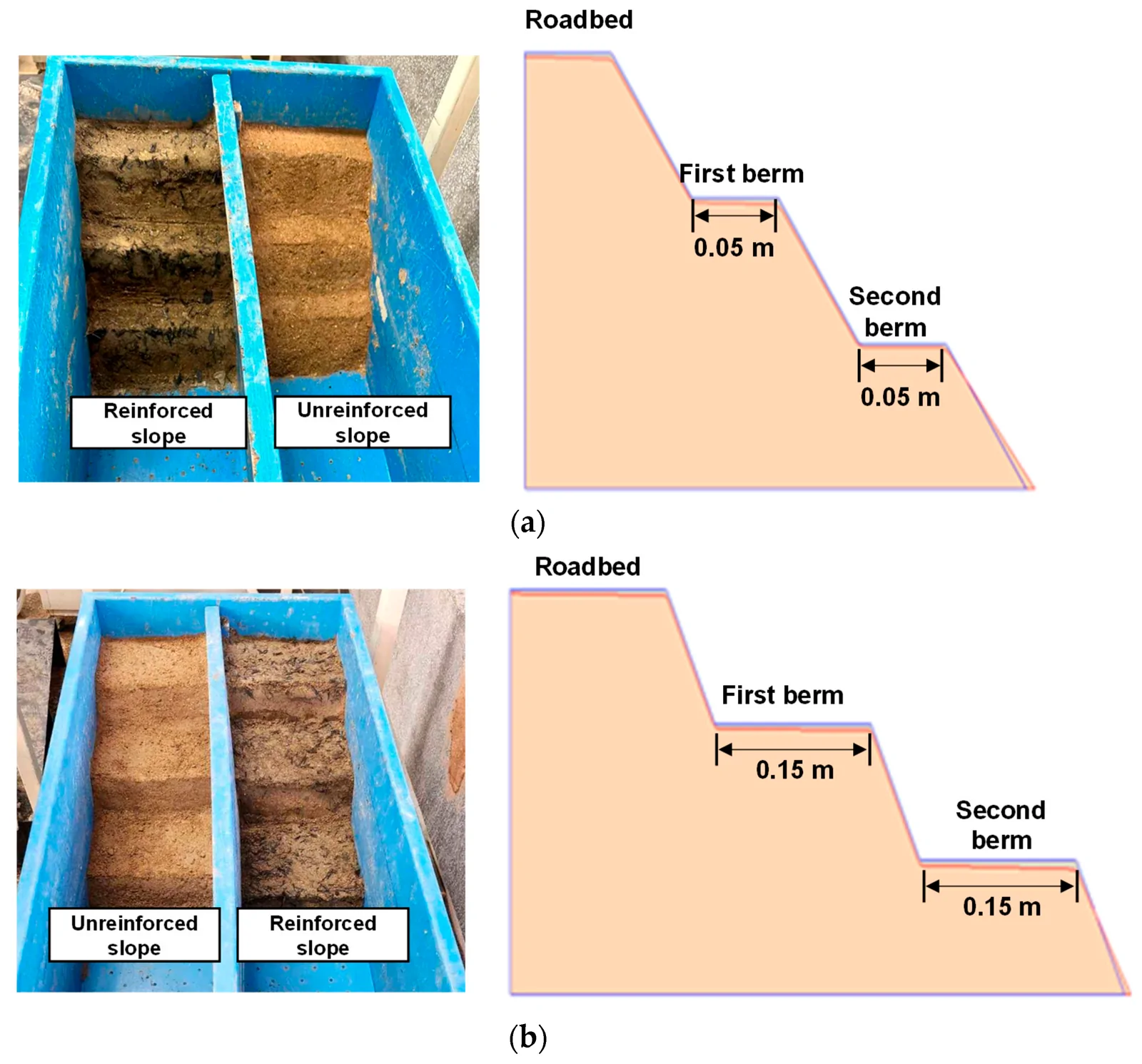
Configuration of the unreinforced and locally reinforced slope models with berm lengths of (**a**) 0.05 m and (**b**) 0.15 m.

The model slopes were geometrically scaled to 1:50 of the prototype and constructed with a slope height of 0.20 m and a slope gradient of 1:1.5. Two berm lengths, 0.05 and 0.15 m, were considered to investigate the influence of berm geometry shown in Figure 6. The experimental conditions for the comparative artificial rainfall model tests are summarized in Table 7. The unreinforced slope was constructed using SW soil with a unit weight of 18.0 kN/m^3^. For the reinforced slope, SW mixed with 20% E.S.B. and 0.6% carbon fiber was compacted at a unit weight of 20.0 kN/m^3^ in the roadbed and the first and second berm regions. An E.S.B.-only control model containing SW with 20% E.S.B. but without carbon fiber was not included in the rainfall-test program. The reinforced material was cured for 7 days prior to rainfall testing. Artificial rainfall with an intensity of 20 mm/h was then applied continuously for 48 h under the same conditions for both reinforced and unreinforced slopes. No unreinforced control model was prepared at a unit weight of 20.0 kN/m^3^. Therefore, the rainfall-model tests compare the overall responses of two configurations that differed in both material composition and initial unit weight, rather than isolating the effect of the stabilization system at an identical unit weight.

Slope behavior was monitored throughout the 48 h rainfall period. Settlement was measured at the roadbed, first berm, and second berm at rainfall durations of 0, 12, 24, 36, and 48 h. Surface erosion and deformation were continuously monitored by close-range photography to identify their initiation and progression. Pore-water pressure and runoff discharge were not directly measured, while surface erosion was evaluated qualitatively through the sequential photographic observations. After completion of the 48 h rainfall test, moisture content was determined at the roadbed, first berm, and second berm. Three measurements were obtained at each location, and their mean value was used for the analysis. One independent rainfall model test was conducted for each slope configuration and berm length. Therefore, the rainfall-test results are presented as individual experimental observations and were not subjected to inferential statistical analysis.

## 3. Results

The experimental results are presented in this section to evaluate the mechanical performance and rainfall resistance of carbon fiber-reinforced stabilized soil. First, the effects of soil type, E.S.B. content, carbon fiber content, and curing period on the unconfined compressive strength are examined to identify an appropriate mixture for slope reinforcement. The shear-strength characteristics of the unreinforced field soil and the selected stabilized soil are then evaluated using direct shear and triaxial compression tests. Finally, the unreinforced and locally stabilized slope models are compared in terms of settlement, final moisture content, and visible surface erosion and deformation during artificial rainfall.

### 3.1. UCS-Based Mixture Selection and Mechanical Behavior

**Figure 7:**
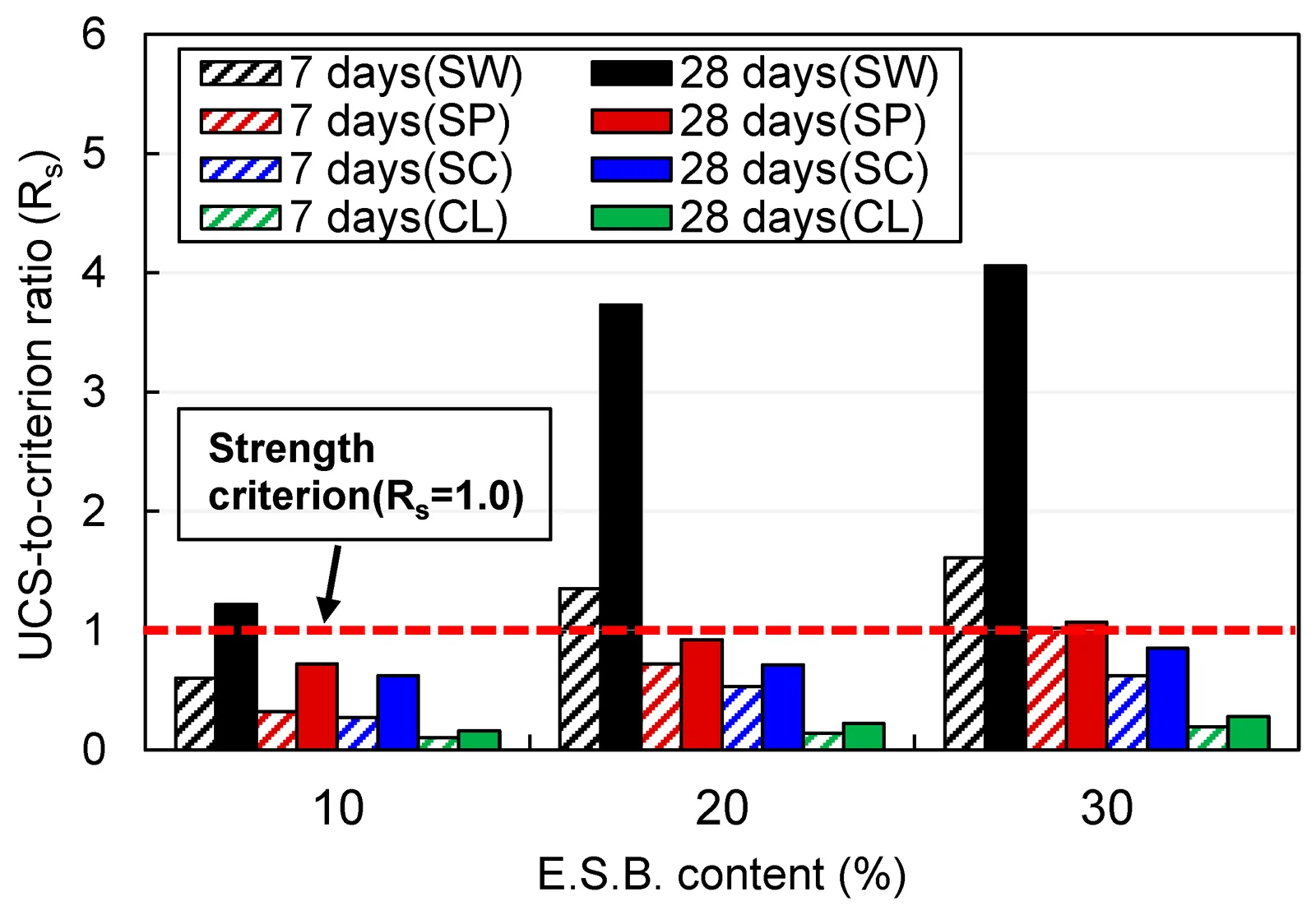
Effect of E.S.B. content and curing period on UCS-to-criterion ratio of different soil types.

Figure 7 compares the UCS-to-criterion ratios of the four soil types at a relative compaction of 95%. Previous guidelines report compressive strength levels of approximately 5.2–6.9 MPa for soil–cement used in base, dam, and embankment protection applications [34,35,36]. Based on these reported values, a 7-day UCS of 6 MPa was adopted in this study as the minimum strength criterion for selecting the stabilized-soil mixture. Accordingly, the UCS-to-criterion ratio is defined as *R_s_* = *q_u_*/*6*, where *q_u_* is the measured UCS in MPa; thus, *R_s_* = 1.0 represents the adopted minimum strength criterion.

The results show that the effectiveness of E.S.B. stabilization was strongly dependent on soil type, E.S.B. content, and curing period. SW exhibited the highest strength development among the four soils. At E.S.B. contents of 20% and 30%, the 7-day strength ratios of SW were 1.35 and 1.61, respectively, and these increased to 3.73 and 4.06 after 28 days of curing. In contrast, SW containing 10% E.S.B. remained below the criterion. SP exhibited substantially lower strength ratios and exceeded the criterion only at 30% E.S.B. after 28 days (*R_s_* = 1.02), whereas SC and CL did not satisfy the criterion under the investigated conditions. These results indicate that SW was the most suitable soil for stabilization. Because 20% E.S.B. provided a substantial strength margin above the adopted criterion, this mixture was selected for the subsequent evaluation of carbon fiber reinforcement.

**Figure 8:**
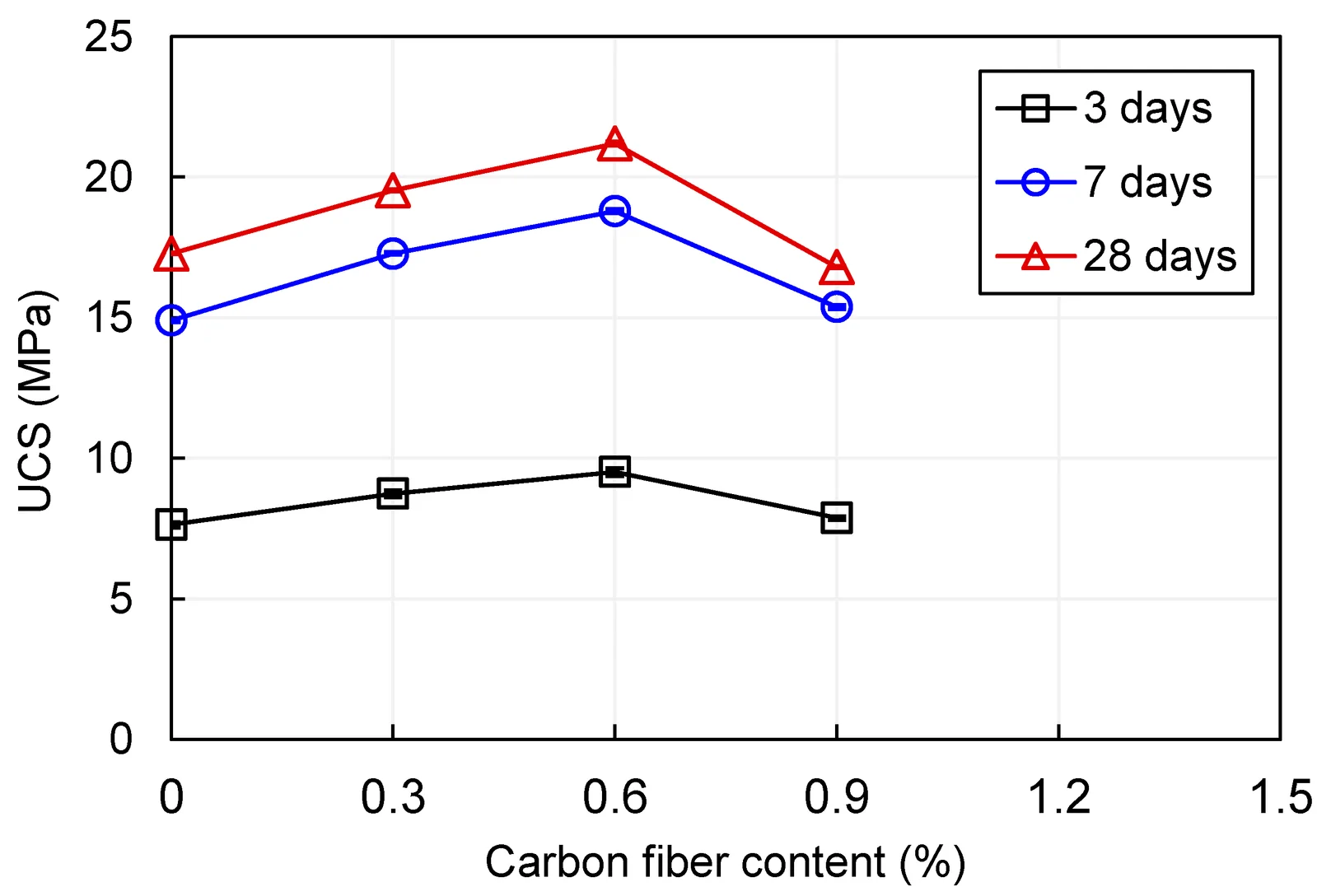
Effect of carbon fiber content and curing period on the unconfined compressive strength (UCS) of SW stabilized with 20% E.S.B. Data are presented as mean ± standard deviation of three replicate specimens (n = 3). Error bars represent ±1 standard deviation (n = 3); in some cases, the error bars are smaller than the plotting symbols.

Figure 8 shows the effect of carbon fiber content and curing period on the UCS of SW stabilized with 20% E.S.B. The UCS increased as the carbon fiber content increased from 0 to 0.6% for all curing periods and subsequently decreased at a fiber content of 0.9%. The maximum UCS was consistently obtained at a carbon fiber content of 0.6%, corresponding to an increase of approximately 22.3–26% compared with the specimens without carbon fiber. In addition, the UCS increased with curing period, and the 7-day strength reached approximately 88–91.5% of the corresponding 28-day strength, indicating that a substantial portion of the long-term strength developed within the first 7 days. These results identify 0.6% as the UCS-optimum carbon fiber content under the tested conditions.

Although specimens containing 1.2% carbon fiber were included in the original test matrix, these data were not plotted in Figure 8 because stable specimens could not be properly formed during specimen preparation, preventing the acquisition of valid UCS results. The original dissertation also reported that excessive fiber incorporation interfered with specimen formation and strength development [37]. Accordingly, the 1.2% condition was excluded from the quantitative UCS comparison because a valid test specimen and corresponding UCS value could not be obtained.

**Figure 9:**
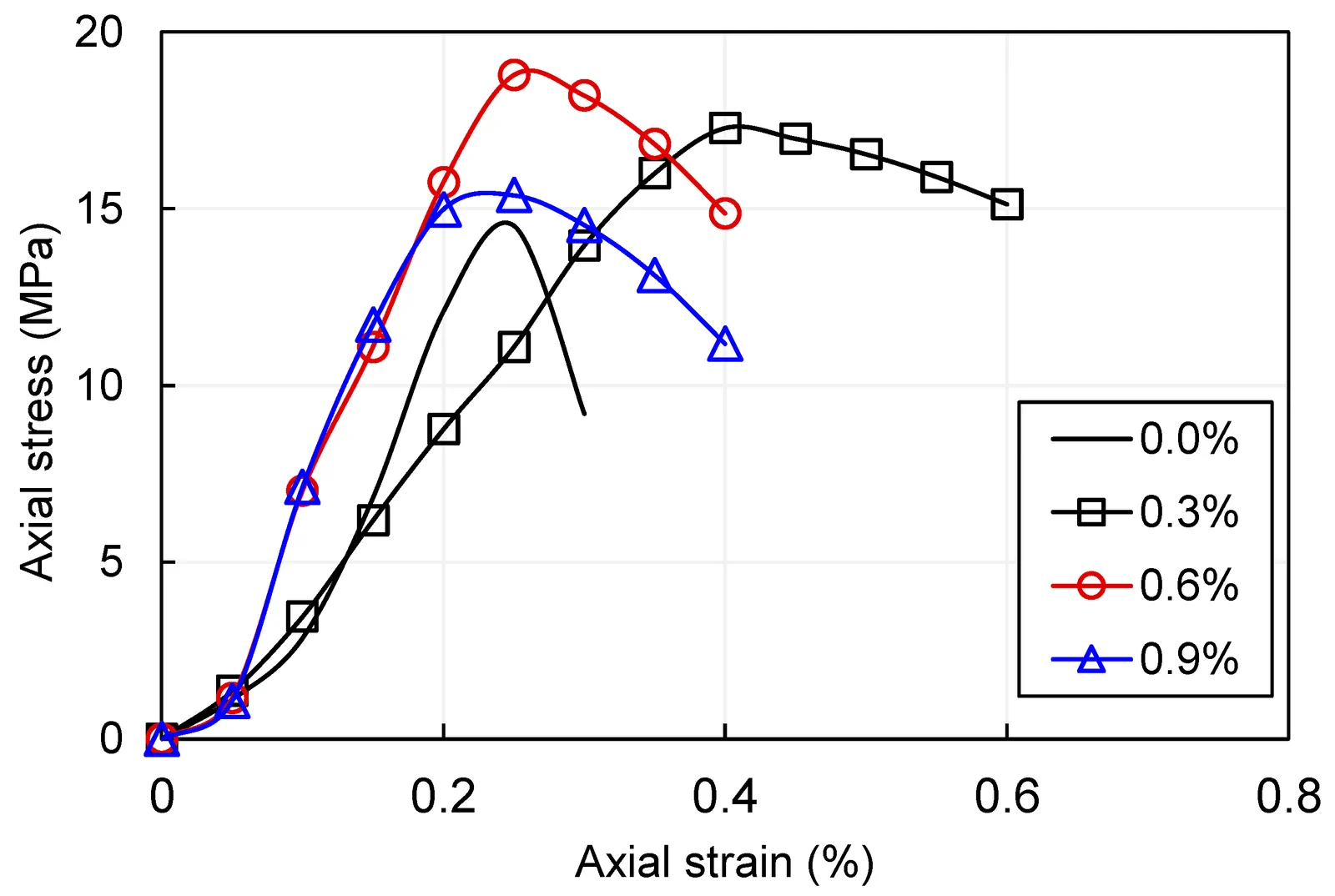
Stress–strain responses of SW stabilized with 20% E.S.B. at different carbon fiber contents after 7 days of curing.

Figure 9 presents the stress–strain responses of SW stabilized with 20% E.S.B. at different carbon fiber contents after 7 days of curing. The stress–strain behavior was markedly influenced by the carbon fiber content. The specimen without carbon fiber exhibited a peak stress of approximately 14–15 MPa, followed by a relatively rapid post-peak reduction. With the addition of carbon fiber, both the peak stress and deformation characteristics changed. The highest peak stress, approximately 19 MPa, was obtained at a carbon fiber content of 0.6%, consistent with the UCS-based selection. In comparison, the specimen containing 0.3% carbon fiber exhibited a lower peak stress but a larger strain at peak and a more gradual post-peak reduction, indicating enhanced deformation capacity.

At a carbon fiber content of 0.9%, the peak stress decreased relative to that at 0.6%, although it remained comparable to or slightly higher than that of the specimen without carbon fiber. The 1.2% fiber condition is not included because stable specimens could not be properly formed, preventing the acquisition of a valid stress–strain response. Overall, the results indicate that carbon fiber addition affects both compressive strength and deformation behavior, with 0.6% providing the highest peak strength under the investigated conditions.

### 3.2. Shear-Strength Characteristics

Table 8 summarizes the shear-strength parameters of the field weathered soil classified as well-graded sand (SW) under different moisture conditions. The friction angle decreased markedly with increasing moisture conditions, from 45.3° in the dry state to 35.8° under the natural moisture condition and further to 30.7° in the saturated state. In contrast, cohesion showed a relatively limited variation, ranging from 0.009 to 0.015 MPa, with the highest value obtained under the natural moisture condition. The saturated soil exhibited the lowest cohesion of 0.009 MPa together with the lowest friction angle. These results demonstrate that the shear resistance of the field soil was reduced as the soil approached saturation, with the most pronounced change occurring in the frictional component of shear strength. The obtained parameters were subsequently used to characterize the shear-strength behavior of the unreinforced slope soil under rainfall-related moisture conditions.

**Figure 10:**
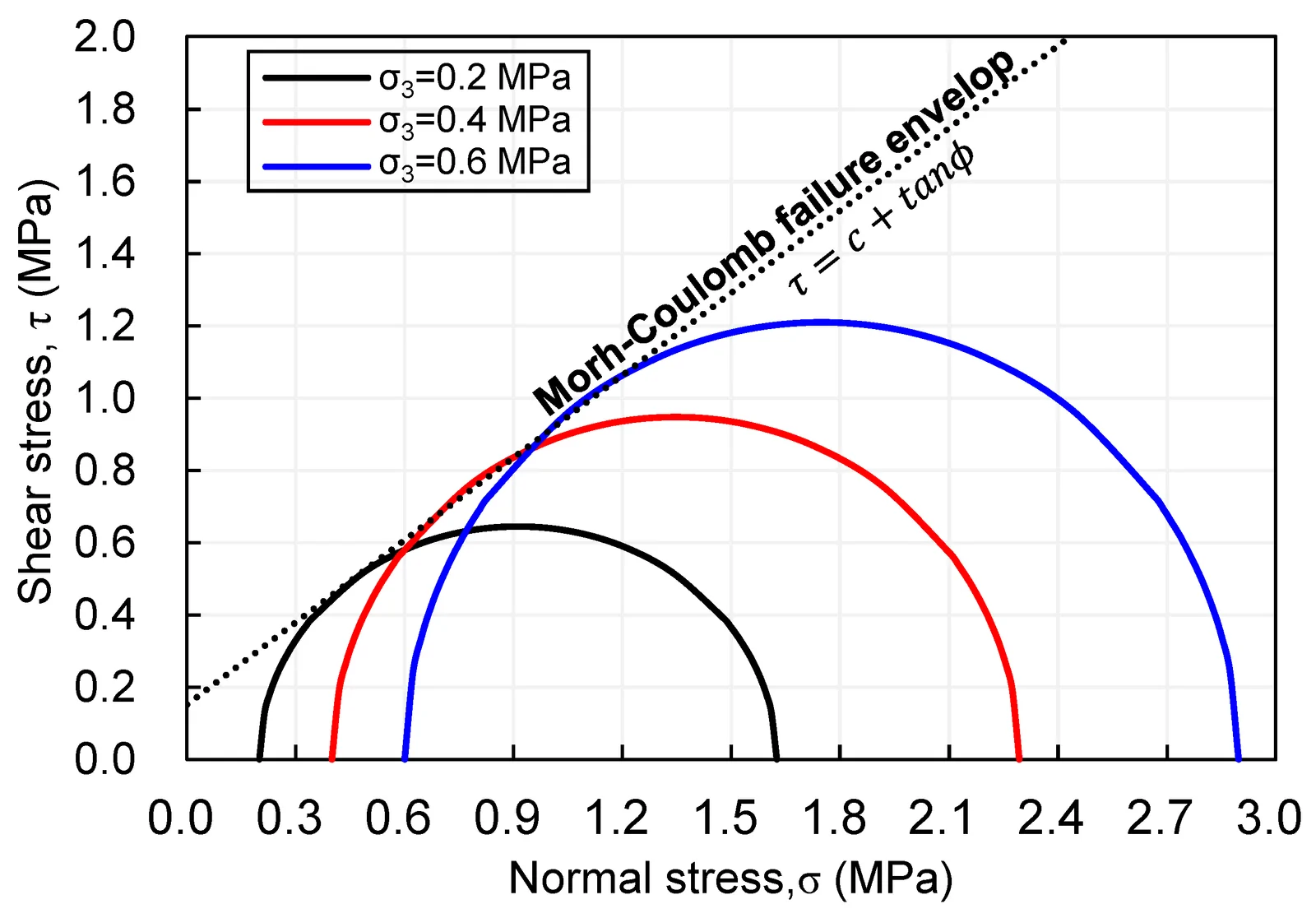
Mohr stress circles and Mohr–Coulomb failure envelope for SW stabilized with 20% E.S.B. and 0.6% carbon fiber.

Table 9 and Figure 10 present the triaxial compression test results for SW stabilized with 20% E.S.B. and 0.6% carbon fiber. As the confining pressure increased from 0.2 to 0.6 MPa, the corresponding Mohr circles increased in size, indicating an increase in the stress required to reach failure under higher confinement. The Mohr–Coulomb failure envelope constructed from the three confining-pressure conditions yielded an apparent cohesion, *c*, of 0.152 MPa and a friction angle, *ϕ*, of 37.23°. These parameters characterize the shear-strength behavior of the selected stabilized mixture under the triaxial test conditions. Because the untreated SW and stabilized mixture were evaluated using different test methods and preparation conditions, their shear-strength parameters are not directly compared quantitatively in this study.

### 3.3. Settlement Behavior Under Rainfall Condition

**Figure 11:**
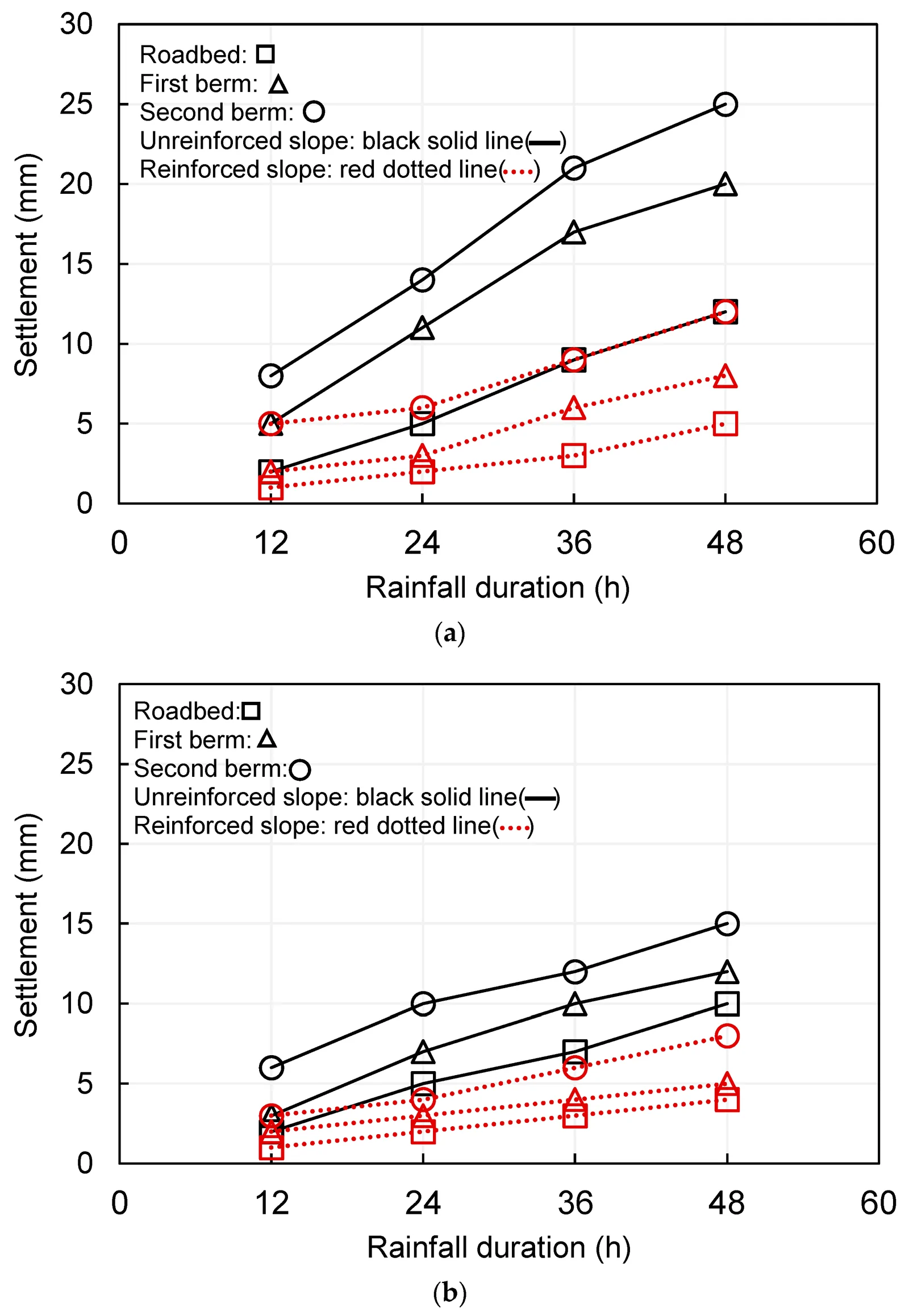
Settlement of unreinforced and reinforced slopes under rainfall with different berm lengths: (**a**) 0.05 m and (**b**) 0.15 m.

Figure 11 compares the settlement behavior of the unreinforced and reinforced model slopes during 48 h of artificial rainfall for berm lengths of 0.05 and 0.15 m. In all cases, settlement progressively increased with rainfall duration, and the largest settlement consistently occurred at the second berm, followed by the first berm and the roadbed. For the 0.05 m berm condition, the settlements of the unreinforced slope after 48 h were 12, 20, and 25 mm at the roadbed, first berm, and second berm, respectively, whereas the corresponding values for the reinforced slope were reduced to 5, 8, and 12 mm. These reductions correspond to approximately 58%, 60%, and 52%, respectively.

A similar trend was observed for the 0.15 m berm. After 48 h of rainfall, settlement decreased from 10, 12, and 15 mm in the unreinforced slope to 4, 5, and 8 mm in the reinforced slope at the roadbed, first berm, and second berm, respectively. The values for the locally stabilized model were approximately 47–60% lower than those for the unreinforced model. These percentages represent descriptive differences between the individual model tests and should not be interpreted as statistically established reductions or as the isolated effects of E.S.B. and carbon fiber because the models were prepared at different initial unit weights. Settlement was also generally lower for the 0.15 m berm than for the 0.05 m berm in both configurations. These reduction ratios represent relative differences observed in the present small-scale model tests and should not be interpreted as quantitative predictions of prototype-scale settlement.

### 3.4. Moisture Content After Rainfall

**Figure 12:**
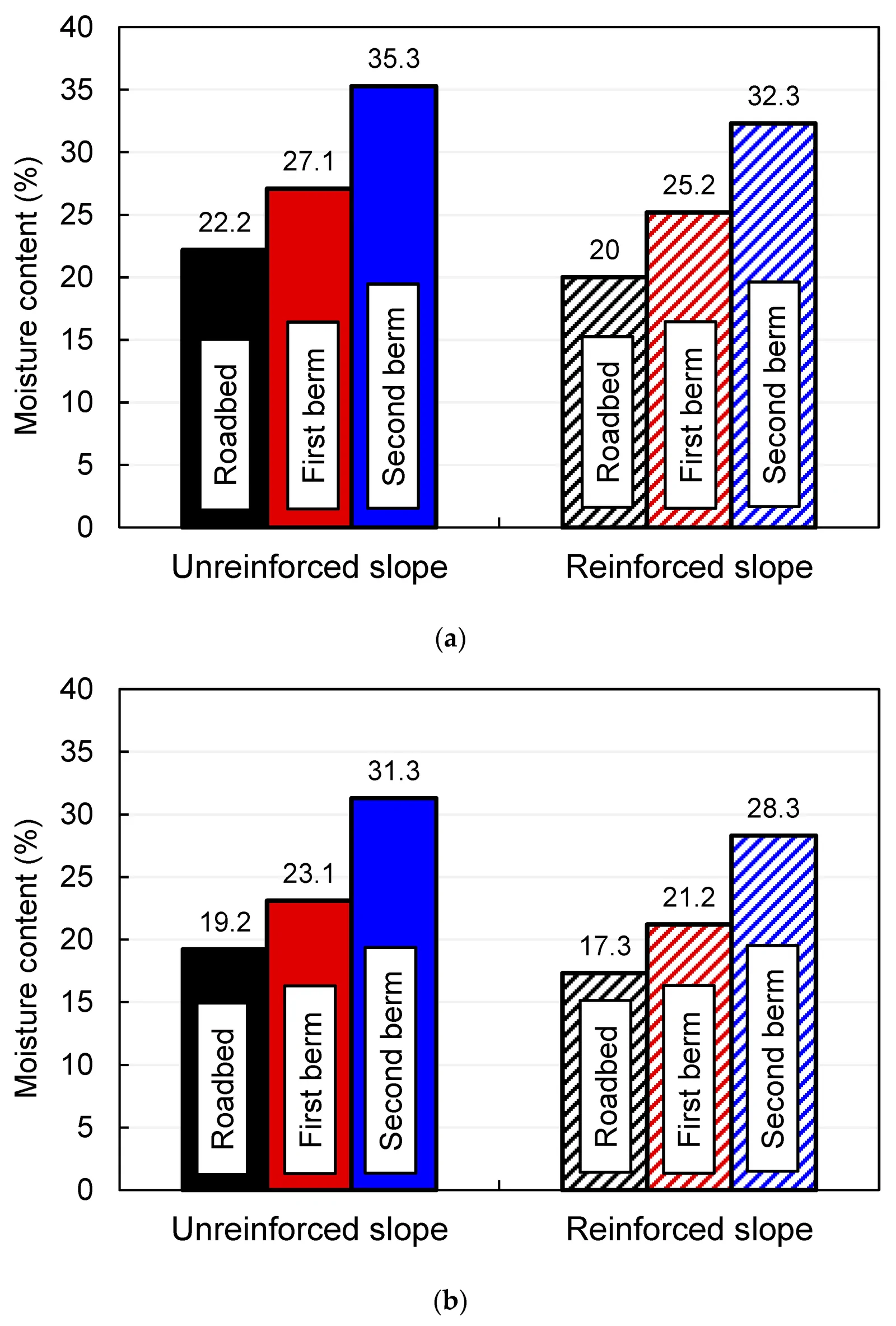
Moisture contents of unreinforced and reinforced slopes after 48 h of rainfall: (**a**) berm length of 0.05 m and (**b**) berm length of 0.15 m.

Figure 12 compares the moisture contents of the unreinforced and reinforced slopes after 48 h of artificial rainfall for berm lengths of 0.05 and 0.15 m. For both berm lengths, the moisture content increased from the roadbed toward the lower part of the slope, with the highest value consistently observed at the second berm. For the 0.05 m berm condition, the moisture contents of the unreinforced slope were 22.2%, 27.1%, and 35.3% at the roadbed, first berm, and second berm, respectively. The corresponding values for the reinforced slope were lower, at 20.0%, 25.2%, and 32.3%, respectively. This indicates that the reinforced slope exhibited lower final moisture contents than the unreinforced slope at all measurement locations after 48 h of rainfall.

A similar trend was observed for the 0.15 m berm condition. The moisture contents of the unreinforced slope were 19.2%, 23.1%, and 31.3% at the roadbed, first berm, and second berm, respectively, whereas those of the reinforced slope were 17.3%, 21.2%, and 28.3%. In addition, the moisture contents measured for the 0.15 m berm were generally lower than those for the 0.05 m berm for both reinforced and unreinforced slopes. Overall, lower final moisture contents were measured in the reinforced slope after 48 h of rainfall, while the second berm exhibited the highest final moisture content regardless of reinforcement condition.

### 3.5. Progressive Erosion and Deformation Under Rainfall

**Figure 13:**
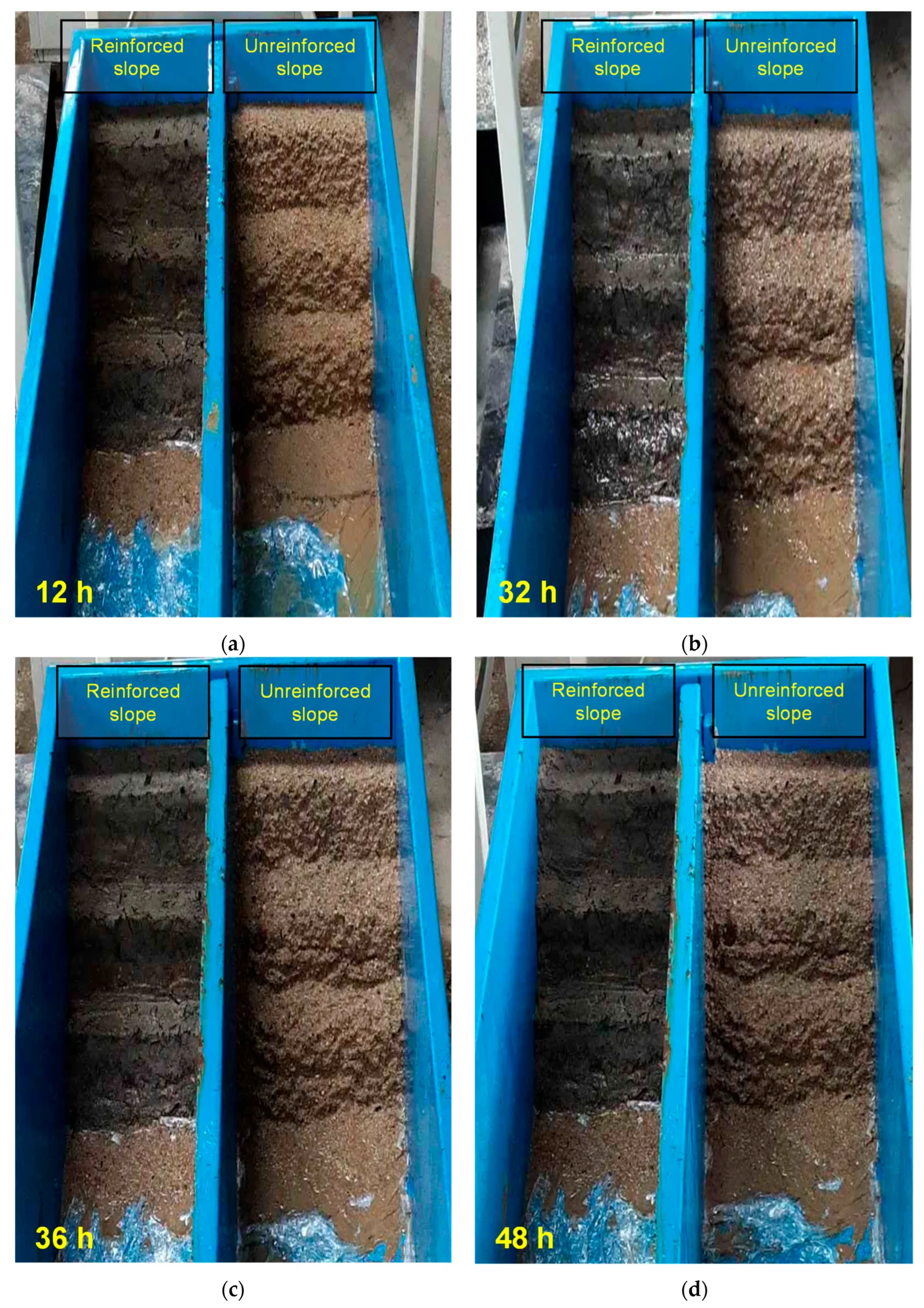
Progressive erosion and deformation of unreinforced and reinforced model slopes during artificial rainfall for a berm length of 0.05 m: (**a**) 12 h, (**b**) 32 h, (**c**) 36 h, and (**d**) 48 h.

Figure 13 shows the progressive erosion and deformation of the unreinforced and reinforced model slopes with a berm length of 0.05 m during artificial rainfall. After 12 h of rainfall, the reinforced slope showed only minor surface changes, whereas initial erosion was already visible on the unreinforced slope. At 32 h, erosion of the unreinforced slope became more pronounced, particularly around the second berm, indicating the onset of localized slope failure. As rainfall continued to 36 h, surface erosion and deformation progressively developed in the unreinforced slope, while the reinforced slope maintained its overall geometry with only minor surface deterioration. After 48 h of continuous rainfall, substantial erosion caused by rainfall and surface runoff was observed in the unreinforced slope, whereas deformation and erosion of the reinforced slope remained limited. These qualitative observations show that the locally stabilized slope exhibited less visible surface erosion and deformation than the unreinforced slope throughout the 48 h rainfall period.

**Figure 14:**
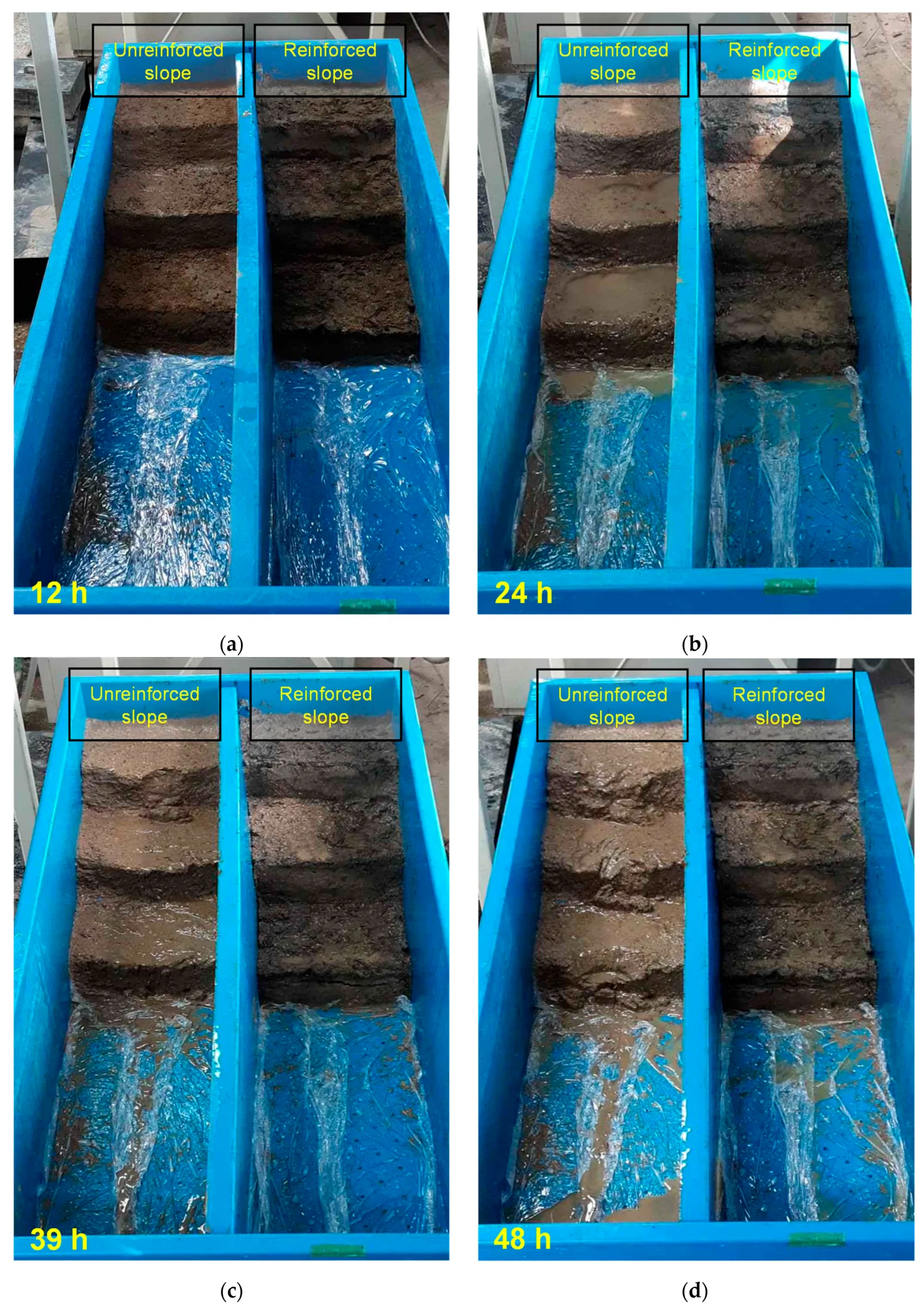
Progressive erosion and deformation of unreinforced and reinforced model slopes during artificial rainfall for a berm length of 0.15 m: (**a**) 12 h, (**b**) 24 h, (**c**) 39 h, and (**d**) 48 h.

Figure 14 shows the progressive erosion and deformation of the unreinforced and reinforced model slopes with a berm length of 0.15 m during artificial rainfall. After 12 h of rainfall, both slopes maintained their overall geometry with only minor surface changes. At 24 h, surface runoff became evident along the roadbed and berm regions, although substantial deformation was not yet observed. As rainfall continued, a clear difference between the two slopes developed. At 39 h, the unreinforced slope exhibited localized shallow failure near the roadbed together with progressive surface erosion, whereas the reinforced slope showed only minor erosion without noticeable deformation. After 48 h of continuous rainfall, erosion and shallow failure had extended over the roadbed and berm regions of the unreinforced slope. In contrast, the reinforced slope largely maintained its original geometry, with no distinct slope deformation observed Overall, qualitative visual observations showed less visible surface erosion and deformation in the locally stabilized slope than in the unreinforced slope over the 48 h rainfall period.

## 4. Discussion

The experimental program evaluated the material-scale mechanical response of the selected stabilized mixture and compared the rainfall-induced behavior of unreinforced and locally stabilized slope models. Because the slope-model configurations differed in both material composition and initial unit weight, the observed slope-scale differences are interpreted as comparative responses rather than as the isolated effects of E.S.B. and carbon fiber. The following discussion examines these relationships and their practical implications and limitations.

### 4.1. Implications for Slope Surface Protection Under Prolonged Rainfall

The principal significance of this study lies in linking the material-scale improvement of carbon fiber-reinforced stabilized soil with its slope-scale performance under prolonged rainfall. Among the investigated mixtures, SW stabilized with 20% E.S.B. and 0.6% carbon fiber showed the most favorable mechanical response. The 0.6% fiber content produced the highest UCS, while the triaxial tests yielded an apparent cohesion of 0.152 MPa and a friction angle of 37.23°.

Recent work by Abbas et al. [38] showed that the early-age compressive and shear-strength development of mixed soil is influenced by material composition and curing conditions, highlighting the importance of early-age mechanical development in stabilized soils. In the present study, the 7-day UCS reached approximately 88–91.5% of the corresponding 28-day strength, indicating substantial early strength development of the selected stabilized mixture.

At a carbon fiber content of 1.2%, stable specimens could not be properly formed during specimen preparation, indicating a practical limitation in workability at the higher fiber content. Since workability was not quantitatively measured, this observation should be interpreted as a specimen-preparation limitation rather than as a quantitative workability threshold.

Previous studies have reported that fiber reinforcement can improve the UCS and ductility of cemented soils [20,21], while carbon fiber has been shown to enhance the mechanical and shear characteristics of soils [24,25]. Wang and Shao [27] also reported improved flexural behavior of carbon-fiber-reinforced soil–cement. These findings are consistent with the mechanical improvement observed in the present study. However, most previous studies have focused primarily on material-scale mechanical behavior, whereas the present study additionally evaluates the selected stabilized mixture through comparative small-scale slope tests under prolonged rainfall.

Because the untreated SW and the stabilized mixture were evaluated using different test methods and preparation conditions, their shear-strength parameters are interpreted independently in this study.

In the artificial rainfall tests, the locally stabilized models exhibited 52–60% lower 48 h settlement for the 0.05 m berm and 47–60% lower settlement for the 0.15 m berm than the unreinforced models. The 0.15 m berm generally exhibited lower settlement and final moisture contents than the 0.05 m berm in both the unreinforced and locally stabilized models. This difference suggests that berm geometry influenced the rainfall response; however, because runoff and pore-water responses were not directly measured, the mechanism responsible for this difference cannot be determined from the present tests. Lower final moisture contents and less visible surface deterioration were also observed in the locally stabilized models. Because transient hydraulic responses were not measured, the lower final moisture contents should not be interpreted as evidence of reduced infiltration or other hydraulic mechanisms. In addition, the unreinforced and locally stabilized models were prepared at unit weights of 18.0 and 20.0 kN/m^3^, respectively. The observed differences therefore reflect the combined influences of material composition and initial unit weight and cannot be attributed exclusively to E.S.B. and carbon fiber. Furthermore, because an E.S.B.-only control model was not included, the independent contribution of carbon fiber to the observed rainfall response cannot be isolated.

The observed behavior may be associated with improved interparticle bonding from hydration and pozzolanic reactions, together with stress transfer and crack bridging by carbon fibers. Although these mechanisms were not directly measured, they are consistent with the reduced rainfall-induced slope deterioration.

### 4.2. Practical Application and Limitations

The results of this study indicate that carbon fiber-reinforced stabilized soil has potential for slope surface protection under prolonged rainfall, particularly at roadbed and berm regions where runoff and localized deformation may develop. The selected mixture of SW, 20% E.S.B., and 0.6% carbon fiber exhibited lower settlement and final moisture contents, together with less visible surface deterioration, in the model tests. However, this mixture should not be regarded as a universal design specification because its performance may vary with soil properties, compaction, slope geometry, rainfall characteristics, and field construction conditions. Another limitation is that the unreinforced and locally stabilized slope models were prepared at different initial unit weights. In addition, an E.S.B.-only control slope was not included; therefore, the independent contribution of carbon fiber to the rainfall response cannot be isolated from the present tests. Future studies should include E.S.B.-only and matched-unit-weight control models.

A major limitation of this study is the use of a 1:50 geometrically scaled model under normal gravity without complete similitude relationships for stress, hydraulic response, soil particle size, and reinforcement dimensions. Therefore, the measured settlement and erosion responses cannot be directly extrapolated to full-scale slopes. This limitation is particularly relevant to carbon fiber reinforcement because the fiber dimensions were not scaled in proportion to the slope geometry. Accordingly, the rainfall tests should be interpreted as comparative small-scale experiments rather than as quantitative simulations of prototype-scale slope behavior.

The rainfall model tests were not independently replicated for each configuration. Consequently, between-test variability could not be quantified, and the observed differences in settlement and moisture content should be interpreted descriptively rather than statistically.

The experimental scope was also limited to a slope gradient of 1:1.5, two berm lengths, and a rainfall intensity of 20 mm/h for 48 h. In addition, the rainfall tests were conducted only for the selected SW-based mixture. Transient pore-water pressure, matric suction, infiltration rate, runoff discharge, and eroded soil mass were not directly measured, limiting quantitative interpretation of the hydraulic mechanisms. The 7-day curing period used for the rainfall-model tests was adopted as a controlled laboratory condition, and the UCS results showed that approximately 88–91.5% of the corresponding 28-day strength had developed by 7 days. However, this laboratory curing condition does not represent field curing variability or long-term durability under repeated wetting–drying and freeze–thaw cycles. Future studies should therefore include independent replicate model tests, full-scale or field-scale tests, broader soil and rainfall conditions, direct hydraulic and erosion measurements, and long-term durability assessments under repeated wetting–drying, freeze–thaw, and environmental exposure.

## 5. Conclusions

This study evaluated the material-scale mechanical properties of carbon fiber-reinforced stabilized soil and compared the rainfall-induced responses of unreinforced and locally stabilized small-scale slope models. Based on the experimental findings, the following conclusions can be drawn:

1. Among the investigated soils, SW exhibited the most favorable stabilization response. At 95% relative compaction, SW containing 20% E.S.B. exceeded the adopted 7-day UCS criterion of 6 MPa and was selected for subsequent carbon fiber reinforcement tests.
2. The UCS-optimum carbon fiber content under the tested conditions was 0.6%. UCS increased with fiber content up to 0.6% and decreased at 0.9%, while stable specimens could not be properly formed at 1.2%, indicating a specimen-preparation limitation at the higher fiber content.
3. The selected mixture of SW, 20% E.S.B., and 0.6% carbon fiber exhibited enhanced mechanical resistance. Triaxial tests yielded an apparent cohesion of 0.152 MPa and a friction angle of 37.23°, together with improved compressive and post-peak behavior.
4. Under the tested small-scale conditions, the locally stabilized models exhibited approximately 52–60% lower 48 h settlement for the 0.05 m berm and 47–60% lower settlement for the 0.15 m berm than the unreinforced models. Because the two configurations differed in initial unit weight, these differences cannot be attributed solely to E.S.B. and carbon fiber.
5. Qualitative visual observations showed less visible surface erosion and localized deformation in the locally stabilized slope models during prolonged rainfall. However, matched-unit-weight control tests and full-scale verification are required before the contribution of the stabilization system can be isolated or applied quantitatively to design.

## Figures and Tables

**Table 1:** Physical properties of the field soils used in this study.

Property	Unit	SW	SP	SC	CL
USCS classification	-	Well-graded sand	Poorly graded sand	Clayey sand	Low-plasticity clay
Maximum dry unit weight	kN/m^3^	21.00	19.20	18.00	15.50
Optimum moisture content	%	14.00	12.00	18.00	40.00
Liquid limit	%	29.30	N/P	33.10	42.00
Plastic limit	%	24.70	N/P	21.90	26.00
Plasticity index	–	4.50	N/P	11.30	16.00
Specific gravity	–	2.65	2.65	2.70	2.65
Coefficient of uniformity	–	10.20	5.90	–	–
Coefficient of curvature	–	1.20	0.80	–	–

**Table 2:** Chemical and physical properties of the Eco Soil Binder (E.S.B.) and cement.

Property	Eco Soil Binder (E.S.B.)	Cement
SiO_2_ (%)	20.07	33.8
Al_2_O_3_ (%)	5.21	12.9
Fe_2_O_3_ (%)	2.96	3.2
CaO (%)	63.08	40.6
MgO (%)	2.67	4.20
SO_3_ (%)	2.97	2.60
Density (g/cm^3^)	3.08	2.84
Specific surface area (cm^2^/g)	3770	2800
Heat of hydration (cal/g)	12.59	39
Initial setting time (h:min)	00:50	04:20
Final setting time (h:min)	01:15	07:45

**Table 3:** Mechanical properties of carbon fiber used in this study [28].

Property	Unit	Value
Number of filaments	–	6K
0° tensile strength	MPa	2900
0° tensile modulus	GPa	140
0° tensile strain	%	1.8
0° flexural modulus	GPa	125
Interlaminar shear strength (ILSS)	MPa	87

**Table 4:** Test matrix for unconfined compression tests.

Evaluation Step	Soil Type	Relative Compaction (%)	E.S.B. Content (%)	Carbon Fiber Content (%)	Carbon Fiber Length (mm)	Curing Period (Days)
Soil/E.S.B. screening	SW, SP, SC, CL	95	10, 20, 30	0.0	-	3, 7, 28
UCS-based fiber content evaluation	SW	95	20	0.0, 0.3, 0.6, 0.9, 1.2	6	3, 7, 28

**Table 5:** Test conditions for direct shear tests.

Soil Type	Soil Condition	Unit Weight (kN/m^3^)	Moisture Content (%)	Normal Stress (MPa)	Shear Rate (mm/min)
SW	Dry	18.4	–	0.2, 0.4, 0.6	0.1
SW	Wet	20.5	9.0	0.2, 0.4, 0.6	0.1
SW	Saturated	21.5	16.6	0.2, 0.4, 0.6	0.1

**Table 6:** Test conditions for triaxial shear tests.

Soil Type	E.S.B Content (%)	Carbon Fiber Content (%)	Confining Pressure (MPa)	Strain Rate (%/min)
SW	20	0.6	0.2, 0.4, 0.6	1

**Table 7:** Experimental conditions for the comparative indoor artificial rainfall model tests.

Slope Type	Slope Gradient	Rainfall Intensity (mm/h)	Berm Length (m)	E.S.B. Content (%)	Carbon Fiber Content (%)	Unit Weight (kN/m^3^)	Reinforcement Position
Unreinforced slope	1:1.5	20	0.05	-	-	18.0	None
			0.15				
Reinforced slope	1:1.5	20	0.05	20	0.6	20.0	Roadbed, first berm, and second berm
			0.15				

**Table 8:** Direct shear test results for soil collected from the target slope.

Soil Type	Condition	Cohesion, *c* (MPa)	Friction Angle, *φ* (°)
SW	Dry	0.010	45.3
SW	Natural	0.015	35.8
SW	Saturated	0.009	30.7

**Table 9:** Shear strength parameters obtained from triaxial compression.

E.S.B. Content (%)	Fiber Content (%)	Apparent Cohesion, *c* (MPa)	Friction Angle, *φ* (°)
20	0.6	0.152	37.23

## Data Availability

The original contributions presented in this study are included in the article. Further inquiries can be directed to the corresponding author.
